# A generic phase between disordered Weyl semimetal and diffusive metal

**DOI:** 10.1038/s41598-017-14760-8

**Published:** 2017-10-30

**Authors:** Ying Su, X. S. Wang, X. R. Wang

**Affiliations:** 1 Physics Department, The Hong Kong University of Science and Technology, Clear Water Bay, Kowloon, Hong Kong; 2 HKUST Shenzhen Research Institute, Shenzhen, 518057 China; 3 School of Microelectronics and Solid-State Electronics, University of Electronic Science and Technology of China, Chengdu, Sichuan 610054 China

## Abstract

Quantum phase transitions of three-dimensional (3D) Weyl semimetals (WSMs) subject to uncorrelated on-site disorder are investigated through quantum conductance calculations and finite-size scaling of localization length. Contrary to previous claims that a direct transition from a WSM to a diffusive metal (DM) occurs, an intermediate phase of Chern insulator (CI) between the two distinct metallic phases should exist due to internode scattering that is comparable to intranode scattering. The critical exponent of localization length is *ν* $\,\simeq$ 1.3 for both the WSM-CI and CI-DM transitions, in the same universality class of 3D Gaussian unitary ensemble of the Anderson localization transition. The CI phase is confirmed by quantized nonzero Hall conductances in the bulk insulating phase established by localization length calculations. The disorder-induced various plateau-plateau transitions in both the WSM and CI phases are observed and explained by the self-consistent Born approximation. Furthermore, we clarify that the occurrence of zero density of states at Weyl nodes is not a good criterion for the disordered WSM, and there is no fundamental principle to support the hypothesis of divergence of localization length at the WSM-DM transition.

## Introduction

Weyl semimetals (WSMs), characterized by the linear crossings of their conduction and valence bands at Weyl nodes (WNs) and the inevitable generation of topologically protected surface states, have attracted enormous attention in recent years because of their exotic properties and possible applications^1–12^. Interestingly, WSM crystals are quite common instead of rare. The reason is that the most generic Hamiltonian describing two bands of a crystal is the direct sum of 2 × 2 matrices in the momentum space as $H={\cup }_{{\boldsymbol{k}}}\oplus h({\boldsymbol{k}})$, where ***k*** is the lattice momentum. Thus, *h*(***k***) must take a form of ${\varepsilon }_{0}({\boldsymbol{k}})I+{\sum }_{\alpha }{h}_{\alpha }({\boldsymbol{k}}){\sigma }_{\alpha }$, where *I*, *σ*_*α*_, and *h*_*α*_ (*α* = *x*, *y*, *z*) are respectively the 2 × 2 identity matrix, Pauli matrices, and functions of ***k*** characterizing materials. The two bands cross each other at a WN of ***k*** = ***K*** when *h*_*α*_(***K***) = 0. This can happen in three dimensions (3D) because three conditions match with three variables, and the level repulsion principle can at most shift the WNs. Moreover, WNs must come in pairs with opposite chirality according to the no-go theorem^13^, and the band inversion occurs between two paired WNs, resulting in the topologically protected surface states and accompanying Fermi arcs on crystal surfaces. The only way to destroy a WSM is the merging of two WNs of opposite chirality or via superconductivity^11^.

How does the above picture based on the lattice translational symmetry change when disorders are presented and the lattice momentum is not a good quantum number anymore? This is an important question that has been investigated intensively with conflicting results^14–31^. Disorder can greatly modify electronic structures, resulting in the well-known Anderson localization. One expects that disorder has much more interesting effects to a WSM than that to a normal metal. For example, electrons with linear dispersion relations around the WNs (Dirac nodes) are governed by the effective Weyl (massless Dirac) equation. Weyl electrons cannot be confined by any potential due to the Klein paradox^32^. Early theoretical studies ignored internode scattering and predicted that the WSM phase featured by vanishing density of states (DOS) at WNs is robust against weak disorder and undergoes a direct quantum phase transition to the diffusive metal (DM) phase as disorder increases^14–19^. The divergence of the bulk state localization length at the WSM-DM transition was conjectured^16,20^ and was used in recent numerical studies^26–28^ to support disordered WSMs in a wide range of disorder and direct WSM-DM transitions. Strangely, the evidences of the transition resemble the conventional Anderson localization transitions at which the localization lengths of different sample sizes cross at the same point, and the uncorrelated on-site disorder is used in these studies so that internode scattering is comparable to intranode scattering and should be significantly important. However, a real WSM has at least two WNs of opposite chirality, and disorder can mix two nodes by internode scattering so that the Anderson localization can happen as shown in the disordered graphene^33^. Therefore, the applicability of the direct WSM-DM transition conjectured by theories of a single WN^14–19^ for real disordered WSMs is questionable. The predicted vanishing DOS at WNs have also attracted many numerical studies^20,25,27,31^, and recent works concluded that zero DOS cannot exist at nonzero disorder due to rare region effects and no WSM phase is allowed at an arbitrary weak disorder if zero DOS at WNs is demanded^21,31^.

Strictly speaking, because the lattice momentum is not a good quantum number in a disordered WSM, ***k***-space is only an approximate language although the concepts of band and DOS are still accurate. Thus, the validity of DOS *ρ*(*E*) ∝ *E* ^2^ from 3D linear dispersion relations as a signature of disordered WSMs is doubtful. The distinct property of a WSM is the existence of topologically protected surface states that do not necessarily rely on the linear crossing of two bands and zero DOS at WNs, and should be robust against disorder, at least against the weak one. Therefore, a disordered WSM is defined as a bulk metal with topologically protected surface states in this work. Since both the WSM and DM are bulk metals, bulk states of them are extended and no theoretical basis supports the hypothesis of the divergence of localization length at the WSM-DM transition. Focusing on the previously proposed quantum critical point between the WSM and DM phases^26–28^, we show that the so-called direct WSM-DM transition actually corresponds to two quantum phase transitions and a narrow Chern insulator (CI) phase (which is also called the 3D quantum anomalous Hall phase in ref.^26^) exists between the two distinct metallic phases. The critical exponent of localization length takes the value of 3D Gaussian unitary ensemble of the conventional Anderson localization transition^34–37^. Nontrivial topological nature of the CI phase is confirmed by Hall conductance calculations that show well-defined quantized plateaus in the bulk insulating phase. Furthermore, the disorder-induced various plateau-plateau transitions between different quantized values of Hall conductance can be well explained by the self-consistent Born approximation (SCBA).

## Results

### Model

In order to compare directly with previous studies, we consider a tight-binding Hamiltonian on a cubic lattice of unity lattice constant that was used in refs^2,26^,

1 $${H}_{0}=\sum _{i}{m}_{z}{c}_{i}^{\dagger }{\sigma }_{z}{c}_{i}-\sum _{i}\,[\frac{{m}_{0}}{2}({c}_{i+\hat{x}}^{\dagger }{\sigma }_{z}{c}_{i}+{c}_{i+\hat{y}}^{\dagger }{\sigma }_{z}{c}_{i})+\frac{t}{2}({c}_{i+\hat{z}}^{\dagger }{\sigma }_{z}{c}_{i}+i{c}_{i+\hat{x}}^{\dagger }{\sigma }_{x}{c}_{i}+i{c}_{i+\hat{y}}^{\dagger }{\sigma }_{y}{c}_{i})+{\rm{H}}{\rm{.c}}{\rm{.}}],$$

where ${c}_{i}^{\dagger }=({c}_{i,\uparrow }^{\dagger },{c}_{i,\downarrow }^{\dagger })$ and *c*_*i*_ are electron creation and annihilation operators at site *i*. $\hat{x}$, $\hat{y}$, $\hat{z}$ are unit lattice vectors in *x*, *y*, *z* direction, respectively. *σ*_*x*,*y*,*z*_ are Pauli matrices for spin. The Hamiltonian Eq. (1) can be block diagonalized in the momentum space as ${{H}}_{0}={\sum }_{{\boldsymbol{k}}}\,{c}_{{\boldsymbol{k}}}^{\dagger }{ {\mathcal H} }_{0}({\boldsymbol{k}}){c}_{{\boldsymbol{k}}}$, where ${ {\mathcal H} }_{0}({\boldsymbol{k}})=({m}_{z}-t\,\cos \,{k}_{z}){\sigma }_{z}-{m}_{0}(\cos \,{k}_{x}+\,\cos \,{k}_{y}){\sigma }_{z}+$ $t(\sin \,{k}_{x}{\sigma }_{x}+\,\sin \,{k}_{y}{\sigma }_{y})$. The dispersion relation of the Hamiltonian is ${\varepsilon }_{\pm }({\boldsymbol{k}})=\pm \sqrt{{\rm{\Delta }}{({\boldsymbol{k}})}^{2}+{t}^{2}({\sin }^{2}{k}_{x}+{\sin }^{2}{k}_{y})}$ with ${\rm{\Delta }}({\boldsymbol{k}})={m}_{z}-t\,\cos \,{k}_{z}-{m}_{0}(\cos \,{k}_{x}+\,\cos \,{k}_{y})$. In this study, ${m}_{0}=2.1t$, identical to that in ref.^26^, is used. Model parameter ${m}_{z}$ is the tunable variable to control different phases. The energy band gap closing requires ${\rm{\Delta }}({\boldsymbol{k}})=0$ at ${k}_{x,y}=0$ or $\pm \pi$ that gives the WSM phases of the model: (1) $2{m}_{0}-t\le {m}_{z}\le 2{m}_{0}+t$ with the WNs located at ${\boldsymbol{k}}=(0,0,\pm {\rm{c}}{\rm{o}}{{\rm{s}}}^{-1}({m}_{z}/t-2{m}_{0}/t))$; (2) $-t-2{m}_{0}\le {m}_{z}\le t-2{m}_{0}$ with the WNs located at ${\boldsymbol{k}}=(\pm \pi ,\pm \pi ,\pm {\cos }^{-1}({m}_{z}/t+2{m}_{0}/t))$; (3) $-t\le {m}_{z}\le t$ with the WNs located at ${\boldsymbol{k}}=(0,\pm \pi ,\pm {\cos }^{-1}({m}_{z}/t))$ and $(\pm \pi ,\mathrm{0,}\pm {\cos }^{-1}({m}_{z}/t))$. Otherwise, the two energy bands are gapped. The conditions of the CI phase of the model are: (1) $t < {m}_{z} < -t+2{m}_{0}$; (2) $t\,-\,2{m}_{0} < {m}_{z} < -t$

In order to study the disorder effect, a spin-resolved on-site disorder is included in the model,

2 $$H={H}_{0}+\sum _{i,\sigma }\,{c}_{i,\sigma }^{\dagger }{V}_{i,\sigma }{c}_{i,\sigma },$$

where $\sigma =\uparrow$ or $\downarrow$ and $\{{V}_{i,\sigma }\}$ are uniformly distributed within $[-W/\mathrm{2,}W/\mathrm{2]}$. Here both *H* and ${H}_{0}$ do not have time-reversal symmetry, and $\overline{{V}_{i,\sigma }}=0$ and $\overline{{V}_{i,\sigma }{V}_{i^{\prime} ,\sigma ^{\prime} }}={W}^{2}{\delta }_{i,i^{\prime} }{\delta }_{\sigma ,\sigma ^{\prime} }/12$ with the bar denoting ensemble average over different configurations. According to the Fermi golden rule, the internode and intranode scattering around the WNs have the same rate of

3 $${{\rm{\Gamma }}}_{{\rm{inter}}}={{\rm{\Gamma }}}_{{\rm{intra}}}=\frac{\pi {W}^{2}\rho ({E}_{F})}{24\hslash },$$

where $\rho ({E}_{F})$ is the DOS at Fermi energy and $\rho \mathrm{(0)}\ne 0$ for nonzero disorder (see methods). Therefore the two kinds of scatterings are equally important in the disordered WSM. Moreover, because $\rho ({E}_{F})$ is an increasing function of $|{E}_{F}|$ around WNs, the scattering rates increases as the Fermi energy shifts away from the WNs.

### Localization length

**Figure 1:**
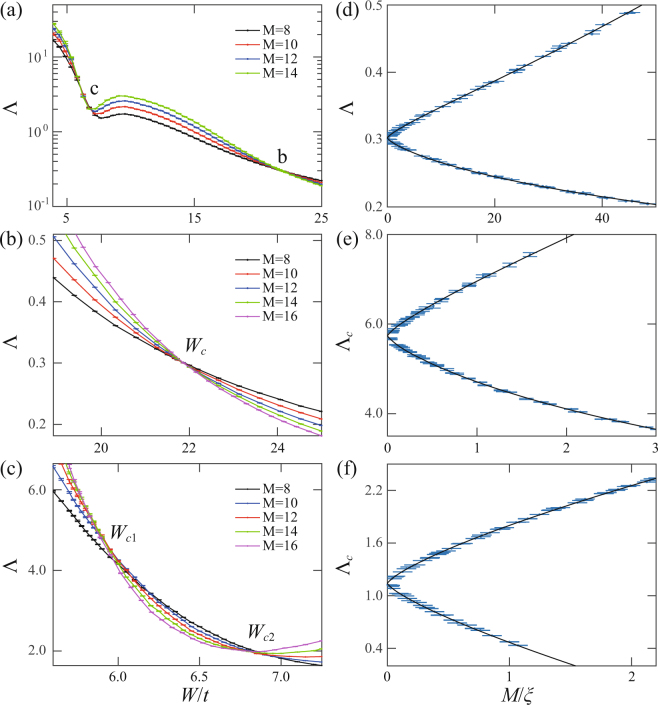
(**a**) The normalized localization length as a function of $W/t$ for various system sizes and with the parameters specified in the text. b and c indicate the possible quantum phase transition points. (**b**,**c**) The close-up shots of the possible transition regions around (**b** and **c**) in (**a**). (**d**) The scaling function obtained by collapsing data points around the critical point ${W}_{{\rm{c}}}$ in (**b**) into the smooth curves. (**e**,**f**) The scaling functions obtained from the corrections to the single-parameter scaling ansatz by collapsing data points around the critical points ${W}_{c1}$ and ${W}_{c2}$ in (**c**) into the smooth curves, respectively.

To investigate various quantum phase transitions in the model, we evaluate the localization length by standard transfer matrix method^38,39^. Here we consider a bar of size ${M}_{x}\times {M}_{y}\times {M}_{z}$ with ${M}_{z}={10}^{5}$ and ${M}_{x}={M}_{y}=M$. Periodic boundary conditions are applied in both $x$ and $y$ directions in order to eliminate surface effects. We fix ${m}_{z}=2.19{m}_{0}$ in the WSM phase since it was reported that the system undergoes a WSM-DM transition as disorder increases^26^. For ${E}_{F}=0$, the normalized localization length ${\rm{\Lambda }}=\lambda (M)/M$ versus $W$ for various $M$ is shown in Fig. 1(a). Very similar to early studies^26–28^, two phase transition points b and c of $d{\rm{\Lambda }}/dM=0$ seem appear. Zooming in on these transition regions, the normalized localization length are shown in Fig. 1(b,c) for b and c, respectively. Apparently, the normalized localization length curves of different $M$ cross at a single critical disorder ${W}_{c}$ in Fig. 1(b) that separates a region of $d{\rm{\Lambda }}/dM > 0$ of a metallic phase for $W < {W}_{c}$ from a region of $d{\rm{\Lambda }}/dM < 0$ of an insulating phase for $W > {W}_{c}$. However, there is a narrow insulating phase characterized by $d{\rm{\Lambda }}/dM < 0$ for ${W}_{c1} < W < {W}_{c2}$ around c, separating two distinct metallic phases ($d{\rm{\Lambda }}/dM > 0$ for $W < {W}_{c1}$ and $W > {W}_{c2}$), as shown in Fig. 1(c).

### Finite-size scaling

**Figure 2:**
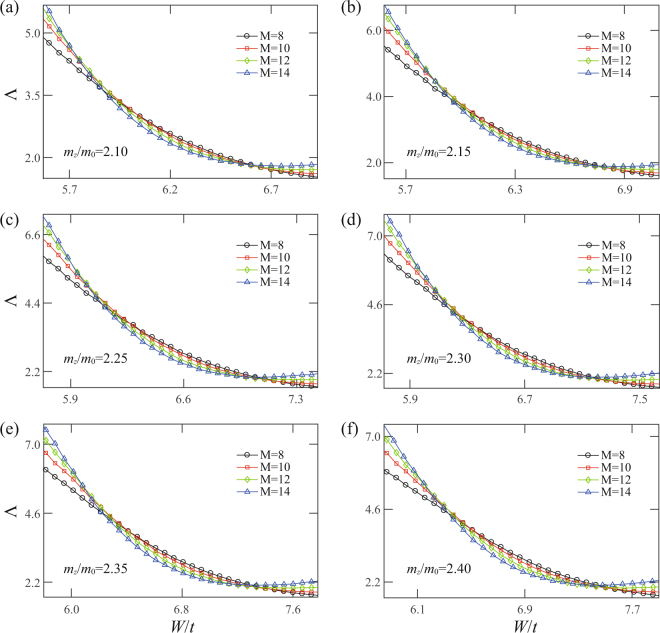
(**a**–**f**) The normalized localization length as a function of $W/t$ for ${m}_{z}/{m}_{0}=2.10$, 2.15, 2.25, 2.30, 2.35, 2.40, respectively. ${E}_{F}=0$ and ${m}_{0}=2.1t$ are fixed.

**Figure 3:**
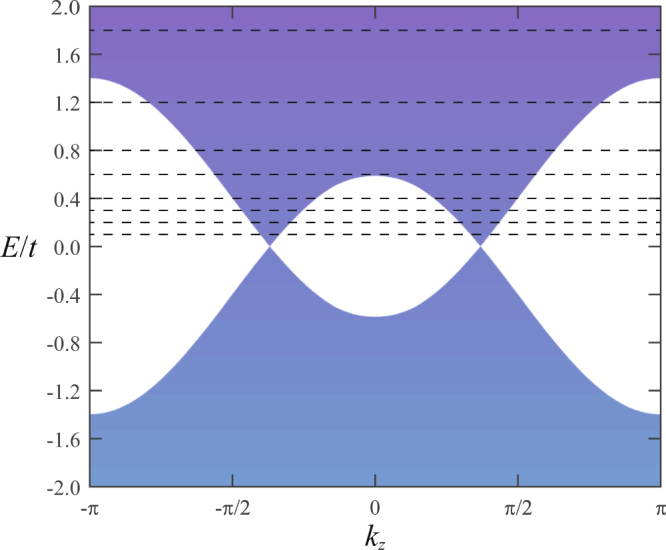
Bulk energy bands of the clean system (with the model parameters ${m}_{z}=2.19{m}_{0}$ and ${m}_{0}=2.1t$) projected onto the ${k}_{z}$ − $E/t$ plane. The dashed lines (from down to up) denotes the Fermi energies ${E}_{F}/t$ = 0.1, 0.2, 0.3, 0.4, 0.6, 0.8, 1.2, 1.8, respectively, that are used for localization length calculations, as shown in Fig. 4.

**Figure 4:**
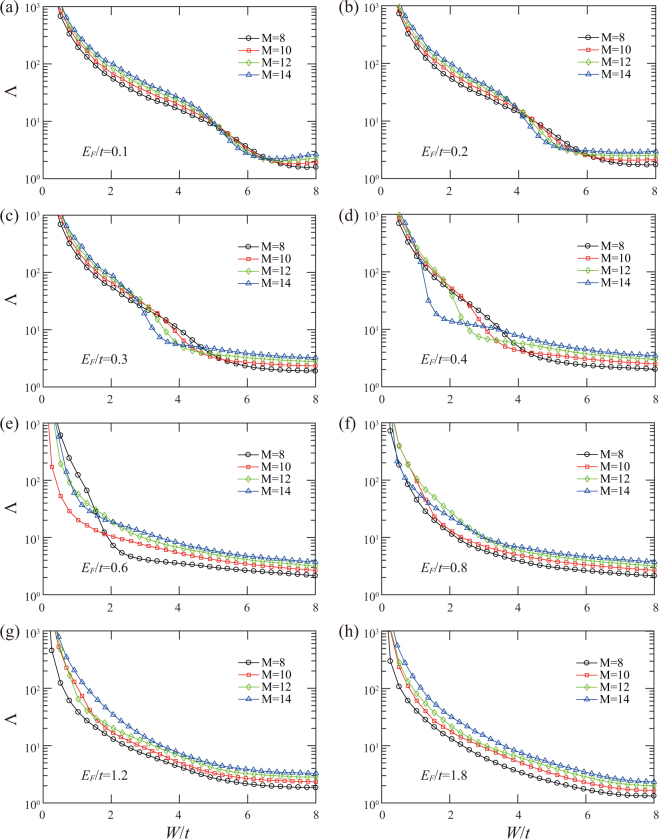
(**a**–**h**) The normalized localization length as a function of $W/t$ for ${E}_{F}/t=0.1$, 0.2, 0.3, 0.4, 0.6, 0.8, 1.2, 1.8, respectively. ${m}_{z}=2.19{m}_{0}$ and ${m}_{0}=2.1t$ are fixed.

To substantiate the criticality of transitions occurring at $W={W}_{c},{W}_{c1},{W}_{c2}$, we employ the finite size scaling analysis for these bulk state localization lengths. For the transition at b, the single-parameter scaling hypothesis is applied as ${\rm{\Lambda }}=f(M/\xi )$, where $\xi  \sim |W-{W}_{c}{|}^{-\nu }$ diverges at the transition point. The scaling functions from both metallic (upper branch) and insulating (lower branch) sides are shown in Fig. 1(d). The perfect collapse of the data points in Fig. 1(b) into the smooth curves supports our claim of the quantum phase transition. The analysis yields ${W}_{c}/t=21.81\pm 0.02$ and $\nu =1.31\pm 0.02$, consistent with the previous numerical and experimental results^34–37^ for 3D Gaussian unitary ensemble. For the quantum phase transitions at critical points ${W}_{c1}$ and ${W}_{c2}$ shown in Fig. 1(c), the crossing of different curves is less perfect as it often happens in 3D systems when the system size is limited by the computer resources. We therefore follow the more accurate analysis used in ref.^40^ to include the contributions of the most important irrelevant parameter to the scaling function

4 $${\rm{\Lambda }}=F(\psi {M}^{\mathrm{1/}\nu },\varphi {M}^{\mu }),$$

where $\psi$ is the relevant scaling variable with $\nu  > 0$ and $\varphi$ is the irrelevant scaling variable with $\mu  < 0$. Using $\nu =1.30$ for the 3D Gaussian unitary class and by minimizing ${\chi }^{2}$, we fit the data points around the two transition points shown in Fig. 1(c) to the scaling function Eq. (4) (see methods). Indeed, the perfect scaling curves in Fig. 1(e,f) with ${W}_{c1}/t=5.81\pm 0.06$ and ${W}_{c2}/t=6.58\pm 0.19$ support our analysis. The chi square of the two fittings are ${\chi }^{2}=78.80$ and $82.49$ with the degrees of freedom ${N}_{d}=86$ and 88 (the number of data points minus the number of fitting parameters), respectively. The reduced chi square of the two cases are ${\chi }_{{\rm{red}}}^{2}={\chi }^{2}/{N}_{d}=0.92$ and 0.94, quite satisfactory numbers. We also calculate the localization length for various ${m}_{z}$ (see Fig. 2) and ${E}_{F}$ (see Figs 3 and 4) in the WSM phase. It is shown that the insulating phase between the two distinct metallic phases is generic, as shown in Fig. 2. A phase diagram is constructed in the ${m}_{z}/{m}_{0}-W/t$ plane for ${E}_{F}=0$ and will be discussed below. As ${E}_{F}$ increases from zero energy (see Fig. 3), the intermediate insulating phase expands initially (see Fig. 4) since the internode scattering rate increases with ${E}_{F}$ according to Eq. (3). Further increase of ${E}_{F}$, the linear dispersion relation fails and the system becomes a conventional 3D metal with Fermi energy deep inside the conduction band, as shown in Fig. 4. Moreover, in a recent work, it was shown that the intermediate CI phase becomes more apparent for tilted WNs^41^. This is consistent with our scattering analysis, since tilting WNs increases the density of states around WNs so that the internode scattering rate is enhanced.

### Quantum transport

**Figure 5:**
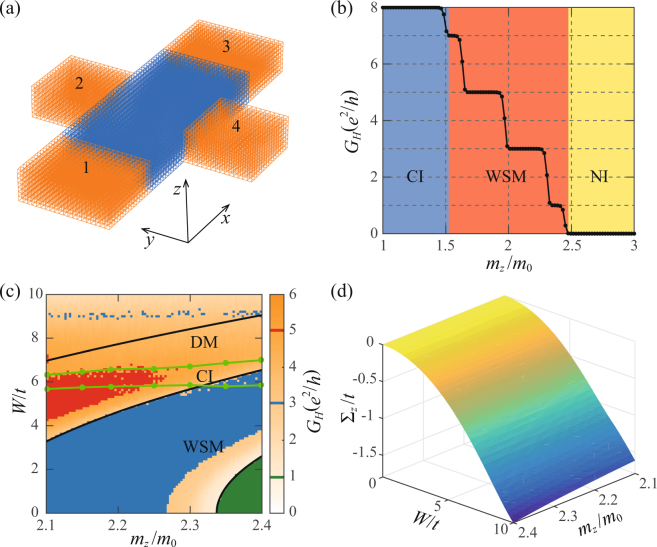
(**a**) The sketch of a four-terminal Hall bar. The blue region is described by the Hamiltonian Eq. (2). The four semi-infinite metallic leads are represented by the orange parts. (**b**) The Hall conductance as a function of ${m}_{z}/{m}_{0}$ for the clean system. The shallow blue, red, and yellow regions mark the CI, WSM, and normal insulator (NI) phases, respectively. (**c**) The density plot of Hall conductance in the ${m}_{z}/{m}_{0}$ − $W/t$ plane for the disordered system. The three black lines (from bottom to top) are plateau-plateau transition lines obtained from the SCBA for $n=1$, 2, 3 in Eq. (7). The two green lines enclose the CI phase region according to the localization length calculations. (**d**) The ${{\rm{\Sigma }}}_{z}$ component of the self-energy obtained from the SCBA as a function of ${m}_{z}/{m}_{0}$ and $W/t$.

In order to investigate the chiral surface states and topological nature of the intermediate insulating phase identified above, we calculate the quantum conductance of a four-terminal Hall bar of size $80\times 40\times 8$ marked by blue color in Fig. 5(a). The bar is described by the Hamiltonian Eq. (2), and the periodic boundary condition is applied in the $z$ direction while the open boundary condition is applied in the $x$ and the $y$ directions. Four semi-infinite metallic leads marked by orange color are connected to the bar as shown in Fig. 5(a). One can view the system as coupled multiple two-dimensional subsystems of ${ {\mathcal H} }_{0}({\boldsymbol{k}})={\sum }_{{k}_{z}}{h}_{{k}_{z}}({k}_{x},{k}_{y})$ with ${k}_{z}=2\pi n/8$, where the integer $n\in [-\mathrm{4,4)}$ labels allowed ${k}_{z}$ within the first Brillouin zone (BZ). For ${k}_{z}\ne {K}_{z}$ (WNs), two-dimensional Hamiltonians ${h}_{{k}_{z}}({k}_{x},{k}_{y})$ are gapped whose Chern number $C({k}_{z})$ is $C(|{k}_{z}| < |{K}_{z}|)=1$ and $C(|{k}_{z}| > |{K}_{z}|)=0$ for $2{m}_{0}-t < {m}_{z} < 2{m}_{0}+t$ ^26^. Thus, a chiral surface state must exist for each allowed ${k}_{z}\in (-|{K}_{z}|,|{K}_{z}|)$, and contribute a quantized Hall conductance of ${e}^{2}/h$. Therefore, the total Hall conductance from the surface states is ${G}_{H}={e}^{2}/h{\sum }_{{k}_{z}}C({k}_{z})={e}^{2}|{K}_{z}|{M}_{z}/(h\pi )$. The Hall conductivity is ${\sigma }_{H}={G}_{H}{M}_{x}/({M}_{y}{M}_{z})={e}^{2}|{K}_{z}|{M}_{x}/(h\pi {M}_{y})$. Moreover, in the CI phase, $C({k}_{z})=\pm 1$ for all the ${k}_{z}$ ^26^. Thus, the Hall conductance is ${G}_{H}=\pm {M}_{z}{e}^{2}/h$.

The Hall conductance in the absence of contact resistance can be calculated from the formula^42^

5 $${G}_{H}\equiv {I}_{13}/{V}_{24}=\frac{{e}^{2}}{h}({T}_{12}-{T}_{14}),$$

where ${T}_{ij}$ is the transmission coefficient from lead $j$ to lead $i$, and current ${I}_{i}$ in lead $i$ is given by the Landauer-Büttiker formalism ${I}_{i}=({e}^{2}/h){\sum }_{j\ne i}({T}_{ji}{V}_{i}-{T}_{ij}{V}_{j})$ where the voltage on lead *i* is ${V}_{i}$ ^43,44^. For the clean system, the Hall conductance as a function of ${m}_{z}/{m}_{0}$ is shown in Fig. 5(b). As expected, the Hall conductances in the normal insulator (NI) and CI phases are respectively $0$ and $8{e}^{2}/h$. In the WSM phase, there are various plateau-plateau transitions between quantized Hall conductances ${G}_{H}\in \mathrm{(0},8{e}^{2}/h)$. Because the change of ${m}_{z}$ shifts WN positions of ${\boldsymbol{k}}=(0,0,\pm {\cos }^{-1}({m}_{z}/t-2{m}_{0}/t))$, the transition from $\mathrm{(2}n+\mathrm{1)}{e}^{2}/h$-plateau to $\mathrm{(2}n-\mathrm{1)}{e}^{2}/h$-plateau occurs whenever ${m}_{z}=2{m}_{0}+t\,\cos \,\mathrm{(2}\pi n/\mathrm{8)}$, where $n=1$, 2, 3 in the current case. The density plot of Hall conductance (ensemble average over 20 configurations) at ${E}_{F}=0$ in the ${m}_{z}/{m}_{0}$ − $W/t$ plane is shown in Fig. 5(c). For ${m}_{z}/{m}_{0}\in \mathrm{[2.1,2.4]}$, the clean system is a WSM whose Hall conduction at WNs is from the surface states and is quantized at a value determined by ${m}_{z}$ as mentioned early. Interestingly, at a fixed ${m}_{z}$ (along a vertical line in Fig. 5(c)), the Hall conductance can jump from one quantized value into another as disorder increases.

### Self-consistent Born approximation

In order to understand these transitions, we use the SCBA to see how the disorder modifies the model parameters^26,45,46^. The self-energy at the Fermi energy due to the disorder is

6 $${\rm{\Sigma }}({m}_{z},W)=\frac{{W}^{2}}{12{S}_{{\rm{BZ}}}}{\int }_{{\rm{BZ}}}\,{d}^{3}{\boldsymbol{k}}{[{E}_{F}+i{0}^{+}- {\mathcal H} ({\boldsymbol{k}},{m}_{z},W)]}^{-1},$$

where ${S}_{{\rm{BZ}}}=8{\pi }^{3}$ is the volume of the first BZ and ${\mathcal H} ({\boldsymbol{k}},{m}_{z},W)={ {\mathcal H} }_{0}({\boldsymbol{k}})+{\rm{\Sigma }}({m}_{z},W)$ is the effective Hamiltonian. For ${E}_{F}=0$, one has ${\rm{\Sigma }}={{\rm{\Sigma }}}_{z}{\sigma }_{z}$ since ${\mathcal H}$ has the particle-hole symmetry^47^. The dispersion relation of the effective Hamiltonian ${\mathcal H}$ is then ${\tilde{\varepsilon }}_{\pm }({\boldsymbol{k}})=\pm \sqrt{{[{\rm{\Delta }}({\boldsymbol{k}})+{{\rm{\Sigma }}}_{z}]}^{2}+{t}^{2}({\sin }^{2}{k}_{x}+{\sin }^{2}{k}_{y})}$. Equation (6) is solved numerically and ${{\rm{\Sigma }}}_{z}({m}_{z},W)$ is shown in Fig. 5(d). Apparently, ${{\rm{\Sigma }}}_{z}({m}_{z},W) < 0$ and is a monotonically decreasing function of *W*. Consequently, the modified mass term ${\tilde{m}}_{z}={m}_{z}+{{\rm{\Sigma }}}_{z}$ decreases and the WNs at ${\boldsymbol{k}}=(0,0,\pm {\cos }^{-1}({\mathop{m}\limits^{ \sim }}_{z}/t-2{m}_{0}/t))$ are shifted towards the BZ boundary as $W$ increases. The plateau-plateau transitions occur at

7 $${\tilde{m}}_{z}({m}_{z},W)=2{m}_{0}+t\,\cos (2\pi n/{M}_{z}),$$

which are plotted as three black curves in Fig. 5(c) for $n=1$, 2, 3 (from bottom to top), respectively. They separate different plateaus. The system becomes a DM at strong disorder (about $W/t > 7$), where the SCBA is not expected to work and no quantized Hall conductance is observed.

### Phase diagram

Our results from localization length and quantum transport calculations are summarized in the phase diagram and the density plot of Hall conductance in the ${m}_{z}/{m}_{0}$ − $W/t$ plane for ${E}_{F}=0$ as shown in Fig. 5(c). Only those ${m}_{z}$, at which the clean system is in the WSM phase and was reported to undergo the WSM-DM transition as disorder increases^26^, are considered. The two green curves are the boundaries of the DM/CI phases (upper line) and CI/WSM phases (lower line). The narrow CI phase region separates the WSM phase from the DM phase. The CI phase is inferred from the fact that all bulk states are localized according to the localization length calculations while the Hall conductance of a finite bar is nonzero and takes several quantized values (red for 5, blue for 3, and green for 1 in units of ${e}^{2}/h$), as shown in Fig. 5(c). The WSM phase is defined as bulk metallic states (extended wavefunctions) with surface conducting channels while the DM phase has bulk metallic states without surface conducting channels. Both the CI and WSM phases can have well quantized Hall conductance (red, blue, and green regions in Fig. 5(c)) while quantized Hall conductance is absent in the DM phase.

## Discussion

The generality of the no direct WSM-DM transition can be understood from the following reasoning. In order to have a direct WSM-DM transition, WNs and topologically protected surface states should be destroyed simultaneously. However, the two events are not exactly the same although they are related. The topologically protected surface states are due to nonzero band Chern numbers of two-dimensional slices between the two WNs. In general, disorder pushes the two WNs away from each other and towards the BZ boundary (as elaborated by the SCBA) where they can merge. As a result, the WNs are destroyed while the nonzero band Chern numbers of two-dimensional slices survive, resulting in the intermediate CI phase. Whether disorder can pull two paired WNs together and towards the BZ center so that the WNs and band Chern numbers can simultaneously be destroyed is an open question.

In conclusion, we show that the claimed direct transition from a WSM to a DM do not exist under uncorrelated on-site disorder due to non-negligible internode scattering. Instead, there exists a intermediate CI phase that separates a WSM phase from a DM phase. Namely, there are actually two quantum phase transitions between the disordered WSM and the DM: One is from the WSM to the CI, and the other is from the CI to the DM. The critical exponent of $\nu \simeq 1.3$ suggests that the two transitions belong to the same universality class of the 3D Gaussian unitary ensemble of the conventional Anderson localization transition. The intermediate CI phase persists and expands at weak disorder as the Fermi energy slightly shifts away from the WNs. Our results do not dependents on specific choices of lattice model since the analysis based on low-energy effective Weyl Hamiltonians is general.

## Methods

### Internode and intranode scattering rates

**Figure 6:**
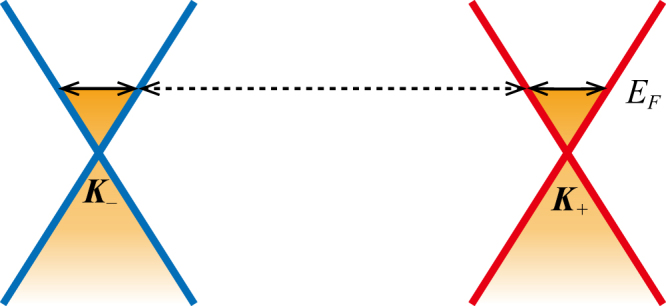
Schematic diagram of internode scattering represented by the dashed arrow and intranode scattering represented by the solid arrows.

The rates of internode and intranode scatterings caused by uncorrelated on-site disorder are derived from low-energy effective Weyl Hamiltonians in this section. For the model parameters ${m}_{z}\in \mathrm{(2}{m}_{0}-t,2{m}_{0}+t)$ studied in the manuscript, the clean system supports a pair of WNs at ${{\boldsymbol{K}}}_{\pm }=\mathrm{(0},0,\pm {\cos }^{-1}({m}_{z}/t-2{m}_{0}/t))$. The low-energy effective Weyl Hamiltonians (to the first order in the momentum deviation ${\boldsymbol{q}}={\boldsymbol{k}}-{{\boldsymbol{K}}}_{\pm }$) around the WNs at ${{\boldsymbol{K}}}_{\pm }$ are

8 $${ {\mathcal H} }_{\pm }({\boldsymbol{q}})=\sum _{\alpha =x,y,z}\,\hslash {v}_{\alpha }^{\pm }{q}_{\alpha }\,{\sigma }_{\alpha },$$

where the Fermi velocities are ${v}_{x}^{\pm }={v}_{y}^{\pm }=t/\hslash$ and ${v}_{z}^{\pm }=\pm \sqrt{{t}^{2}-{({m}_{z}-2{m}_{0})}^{2}}/\hslash$. The energy bands of the Weyl Hamiltonians are $\pm {E}_{q}=\pm \sqrt{{\sum }_{\alpha }{\hslash }^{2}{v}_{\alpha }^{\pm 2}{q}_{\alpha }^{2}}$. To be concrete and without losing generality, we fix the Fermi energy in the conduction band ${E}_{F}={E}_{q}$ as shown in Fig. 6, and the eigenstates with the Fermi energy are

9 $$|c,{{\boldsymbol{K}}}_{\pm }+{\boldsymbol{q}}\rangle =(\begin{array}{c}{a}_{{\boldsymbol{q}}}^{\pm }\\ {b}_{{\boldsymbol{q}}}^{\pm }{e}^{i{\varphi }_{{\boldsymbol{q}}}^{\pm }}\end{array}),$$

where ${a}_{{\boldsymbol{q}}}^{\pm }=\,\cos ({\theta }_{{\boldsymbol{q}}}^{\pm }/\mathrm{2)}$, ${b}_{{\boldsymbol{q}}}^{\pm }=\,\sin ({\theta }_{{\boldsymbol{q}}}^{\pm }/\mathrm{2)}$, $\cos \,{\theta }_{{\boldsymbol{q}}}^{\pm }=\hslash {v}_{z}^{\pm }{q}_{z}/{E}_{F}$, and $\tan \,{\varphi }_{{\boldsymbol{q}}}^{\pm }={v}_{y}^{\pm }{q}_{y}/{v}_{x}^{\pm }{q}_{x}$. In the presence of disorder, the total scattering processes consist of two parts: the internode scattering and intranode scattering that are schematically shown in Fig. 6. According to the Fermi golden rule, the internode and intranode scattering rates are

10 $${{\rm{\Gamma }}}_{{\rm{inter}}}=\sum _{{\boldsymbol{q}}^{\prime} }\,\frac{2\pi }{\hslash }\overline{{|\langle c,{{\boldsymbol{K}}}_{\mp }+{\boldsymbol{q}}^{\prime} |V|c,{{\boldsymbol{K}}}_{\pm }+{\boldsymbol{q}}\rangle |}^{2}}\delta ({E}_{{\boldsymbol{q}}^{\prime} }-{E}_{F})=\frac{\pi {W}^{2}\rho ({E}_{F})}{24\hslash },$$

11 $${{\rm{\Gamma }}}_{{\rm{intra}}}=\sum _{{\boldsymbol{q}}^{\prime} }\,\frac{2\pi }{\hslash }\overline{{|\langle c,{{\boldsymbol{K}}}_{\pm }+{\boldsymbol{q}}^{\prime} |V|c,{{\boldsymbol{K}}}_{\pm }+{\boldsymbol{q}}\rangle |}^{2}}\delta ({E}_{{\boldsymbol{q}}^{\prime} }-{E}_{F})=\frac{\pi {W}^{2}\rho ({E}_{F})}{24\hslash }\mathrm{.}$$

where $V={\sum }_{i,\sigma }{c}_{i,\sigma }^{\dagger }{V}_{i,\sigma }{c}_{i,\sigma }$ is the uncorrelated on-site disorder in Eq. (2) and the bar denotes ensemble average over different configurations. Therefore, we can conclude that the internode and intranode scattering rates are identical in Weyl semimetals subject to uncorrelated on-site disorder. Moreover, the scattering rates increase with $|{E}_{F}|$ since the density of states is an increasing function of $|{E}_{F}|$.

### Correction to the single-parameter scaling hypothesis

Following the more accurate analysis used in ref.^40^ to include the contributions from the most important irrelevant parameter, the scaling function becomes Eq. (4). Under the Taylor expansion around a transition point ${W}_{c}$, the scaling function is

12 $${\rm{\Lambda }}=\sum _{n=0}^{{n}_{I}}\,{\varphi }^{n}{M}^{n\mu }{F}_{n}(\psi {M}^{\mathrm{1/}\nu }),\quad {F}_{n}(\psi {M}^{\mathrm{1/}\nu })=\sum _{m=0}^{{n}_{R}}\,{\psi }^{m}{M}^{m/\nu }{F}_{nm},$$

where $\psi =b(W-{W}_{c})$ and $\varphi ={c}_{0}+{c}_{1}(W-{W}_{c})$ up to the first order^40^. One can remove the contributions from the irrelevant scaling variable to Λ and define the corrected localization length as

13 $${{\rm{\Lambda }}}_{c}={\rm{\Lambda }}-\sum _{n=1}^{{n}_{I}}\,{\varphi }^{n}{M}^{n\mu }{F}_{n}(\psi {M}^{\mathrm{1/}\nu }\mathrm{).}$$

Then, the corrected localization length follows the scaling law

14 $${{\rm{\Lambda }}}_{c}=f(M/\xi ),\quad \xi \sim {|W-{W}_{c}|}^{-\nu }.$$

In our analysis, we choose ${n}_{I}={n}_{R}=2$ and ${F}_{01}={F}_{10}=1$ ^40^.

### Data availability

All data generated or analysed during this study are included in this published article.

## References

[1] Wan X, Turner AM, Vishwanath A, Savrasov SY (2011). Topological semimetal and Fermi-arc surface states in the electronic structure of pyrochlore iridates. Phys. Rev. B.

[2] Yang KY, Lu YM, Ran Y (2011). Quantum Hall effects in a Weyl semimetal: Possible application in pyrochlore iridates. Phys. Rev. B.

[3] Burkov AA, Balents L (2011). Weyl semimetal in a topological insulator multilayer. Phys. Rev. Lett..

[4] Turner, A. M. & Vishwanath, A. Beyond band insulators: topology of semi-metals and interacting phases. Preprint at http://arxiv.org/abs/1301.0330 (2013).

[5] Weng H, Fang C, Fang Z, Bernevig BA, Dai X (2015). Weyl semimetal phase in noncentrosymmetric transition-metal monophosphides. Phys. Rev. X.

[6] Huang SM (2015). A Weyl fermion semimetal with surface Fermi arcs in the transition metal monopnictide TaAs class. Nat. Commun..

[7] Xu SY (2015). Discovery of a Weyl fermion semimetal and topological Fermi arcs. Science.

[8] Lv BQ (2015). Experimental discovery of Weyl semimetal TaAs. Phys. Rev. X.

[9] Lu L (2015). Experimental observation of Weyl points. Science.

[10] Shekhar C (2015). Extremely large magnetoresistance and ultrahigh mobility in the topological Weyl semimetal NbP. Nat. Phys..

[11] Hosur P, Qi XL (2013). Recent developments in transport phenomena in Weyl semimetals. C. R. Phys..

[12] Burkov A (2015). Chiral anomaly without relativity. Science.

[13] Nielsen HB, Ninomiya M (1983). The Adler–Bell–Jackiw anomaly and Weyl fermions in a crystal. Phys. Lett. B.

[14] Fradkin E (1986). Critical behavior of disordered degenerate semiconductors. I. Models, symmetries, and formalism. Phys. Rev. B.

[15] Fradkin E (1986). Critical behavior of disordered degenerate semiconductors. II. Spectrum and transport properties in mean-field theory. Phys. Rev. B.

[16] Goswami P, Chakravarty S (2011). Quantum criticality between topological and band insulators in 3 + 1 dimensions. Phys. Rev. Lett..

[17] Sbierski B, Pohl G, Bergholtz EJ, Brouwer PW (2014). Quantum transport of disordered Weyl semimetals at the nodal point. Phys. Rev. Lett..

[18] Syzranov SV, Radzihovsky L, Gurarie V (2015). Critical transport in weakly disordered semiconductors and semimetals. Phys. Rev. Lett..

[19] Syzranov, S. V. & Radzihovsky, L. High-dimensional disorder-driven phenomena in Weyl semimetals, semiconductors and related systems. Preprint at https://arxiv.org/abs/1609.05694 (2016).

[20] Kobayashi K, Ohtsuki T, Imura KI, Herbut IF (2014). Density of states scaling at the semimetal to metal transition in three dimensional topological insulators. Phys. Rev. Lett..

[21] Nandkishore R, Huse DA, Sondhi SL (2014). Rare region effects dominate weakly disordered three-dimensional Dirac points. Phys. Rev. B.

[22] Zhao YX, Wang ZD (2015). Disordered Weyl semimetals and their topological family. Phys. Rev. Lett..

[23] Altland A, Bagrets D (2015). Effective field theory of the disordered Weyl semimetal. Phys. Rev. Lett..

[24] Altland A, Bagrets D (2016). Theory of the strongly disordered Weyl semimetal. Phys. Rev. B.

[25] Pixley JH, Goswami P, Das Sarma S (2015). Anderson localization and the quantum phase diagram of three dimensional disordered dirac semimetals. Phys. Rev. Lett..

[26] Chen CZ (2015). Disorder and metal-insulator transitions in Weyl semimetals. Phys. Rev. Lett..

[27] Liu S, Ohtsuki T, Shindou R (2016). Effect of disorder in a three-dimensional layered Chern insulator. Phys. Rev. Lett..

[28] Shapourian H, Hughes TL (2016). Phase diagrams of disordered Weyl semimetals. Phys. Rev. B.

[29] Bera S, Sau JD, Roy B (2016). Dirty Weyl semimetals: Stability, phase transition, and quantum criticality. Phys. Rev. B.

[30] Roy B, Juricic V, Das Sarma S (2016). Universal optical conductivity of a disordered Weyl semimetal. Sci. Rep..

[31] Pixley JH, Huse DA, Das Sarma S (2016). Rare-region-induced avoided quantum criticality in disordered three-dimensional Dirac and Weyl semimetals. Phys. Rev. X.

[32] Klein O (1929). Reflexion von Elektronen an einem Potentialsprung nach der relativistischen Dynamik von Dirac. Z. Phys..

[33] Zhang, Y. Y. *et al*. Localization and the Kosterlitz-Thouless transition in disordered graphene. *Phys*. *Rev*. *Lett*. **102**, 106401, and references therein (2009). 10.1103/PhysRevLett.102.106401 19392133

[34] Stupp H, Hornung M, Lakner M, Madel O, Löhneysen HV (1993). Possible solution of the conductivity exponent puzzle for the metal-insulator transition in heavily doped uncompensated semiconductors. Phys. Rev. Lett..

[35] Hofstetter E, Schreiber M (1994). Does broken time reversal symmetry modify the critical behavior at the metal-insulator transition in 3-dimensional disordered systems?. Phys. Rev. Lett..

[36] Kawarabayashi T, Ohtsuki T, Slevin K, Ono Y (1996). Anderson transition in three-dimensional disordered systems with symplectic symmetry. Phys. Rev. Lett..

[37] Hofstetter E (1998). Disordered systems and the metal-insulator transition: A super universality class. Phys. Rev. B.

[38] Kramer B, Mackinnon A (1993). Localization: theory and experiment. Rep. Prog. Phys..

[39] Xie XC, Wang XR, Liu DZ (1998). Kosterlitz-Thouless-type metal-insulator transition of a 2D electron gas in a random magnetic field. Phys. Rev. Lett..

[40] Slevin K, Ohtsuki T (1999). Corrections to scaling at the Anderson transition. Phys. Rev. Lett..

[41] Wu YJ, Liu HW, Jiang H, Xie XC (2017). Global phase diagram of disordered type-II Weyl semimetals. Phys. Rev. B.

[42] Sheng L, Sheng DN, Ting CS, Haldane FDM (2005). Nondissipative spin Hall effect via quantized edge transport. Phys. Rev. Lett..

[43] Landauer R (1957). Spatial variation of currents and fields due to localized scatterers in metallic conduction. IBM J. Res. Dev..

[44] Büttiker M (1986). Four-terminal phase-coherent conductance. Phys. Rev. Lett..

[45] Groth CW, Wimmer M, Akhmerov AR, Tworzyd lo J, Beenakker CWJ (2009). Theory of the topological Anderson insulator. Phys. Rev. Lett..

[46] Su Y, Avishai Y, Wang XR (2016). Topological Anderson insulators in systems without time-reversal symmetry. Phys. Rev. B.

[47] Hermanns M, O’Brien K, Trebst S (2015). Weyl spin liquids. Phys. Rev. Lett..

